# Prognostic value of CSN5 in patients with digestive system cancers: a systematic review and meta-analysis

**DOI:** 10.1186/s12885-022-09867-9

**Published:** 2022-07-23

**Authors:** Yonghua Guo, Meng Gao, Ye Yao, Jinghua Li, Xi Chen, Xingxing Wang, Zhang Chen, Yufeng Yuan, Weijie Ma

**Affiliations:** 1 Department of Hepatobiliary and Pancreatic Surgery, Zhongnan Hospital of Wuhan University, Hubei, PR China; 2 Clinical Medicine Research Center for Minimally Invasive Procedure of Hepatobiliary & Pancreatic Diseases of Hubei Province, Hubei, PR China

**Keywords:** Jab1, CSN5, Cancer, Digestive system, Meta-analysis

## Abstract

**Background:**

Despite the understanding of the COP9 signalosome subunit 5 (CSN5) in tumor genesis, there is no conclusive evidence on its value to predict the survival and prognosis of digestive system tumor patients. Hence this study aimed to evaluate the impact of CSN5 levels on the survival and clinicopathological parameters of digestive system neoplasm patients.

**Methods:**

First, a comprehensive search was conducted in four databases. We utilized the Hazard Ratio (HR) with a 95% confidence interval (CI) to evaluate the prognostic value of CSN5 for the overall survival (OS) and recurrence-free survival (RFS) of patients. Then, we estimated the connection between CSN5 and the clinicopathological parameters based on the Odds Ratio (OR) with the corresponding 95% CI.

**Results:**

This meta-analysis included 22 studies and 2193 patients diagnosed with digestive system tumors. High expression of CSN5 was correlated to poorer OS (HR = 2.28, 95% CI: 1.71–3.03; *p* < 0.00001). Additionally, high CSN5 levels were correlated with worse invasion depth (OR = 0.49, 95% CI: 0.25–0.96, *p* = 0.04), positive lymphatic metastasis (OR = 0.28, 95% CI: 0.16–0.47, *p* = 0.00001), positive distant metastasis (OR = 0.32, 95% CI: 0.13–0.76, *p* = 0.01) and poorer differentiation degree (OR = 0.34, 95% CI: 0.19–0.60, *p* = 0.0003). However, we did not detect a correlation between CSN5 expression and age, gender, tumor stage, tumor size or vascular invasion. Furthermore, no significant publication bias was detected.

**Conclusion:**

This meta-analysis demonstrated that the overexpression of CSN5 level might foresee poorer OS in digestive system cancer patients. Additionally, CSN5 levels might be related to the prognosis of digestive system tumors.

**Supplementary Information:**

The online version contains supplementary material available at 10.1186/s12885-022-09867-9.

## Background

Digestive system neoplasm is regarded as a malignancy with high morbidity worldwide, with almost 6.1 million new cases diagnosed annually [1]. Despite the recent progress in clinical medicine, many patients with tumors are still diagnosed at advanced stages, and, if early-stage patients can be identified and treated before tumor deterioration, the mortality would diminish. Therefore, to achieve early detection and intervention of digestive system tumors, reliable diagnostic and prognostic markers with high sensitivity and specificity are required.

The C-Jun activation binding protein (Jab1) interacts with C-Jun at its activation domain and increases the stability of C-Jun or Jun D complexes acting on AP-1 transcription factor binding sites, specifically improving the activation of downstream genes [2]. Besides, Jab1 is also known as the COP9 signalosome subunit 5 (CSN5) [3], participating in the regulation of signal transduction [4–6], cellular proliferation [7] and cell apoptosis [8], as well as deregulation of genomic stability and DNA repair [9]. One of the most important functions of Jab1/CSN5 is to regulate protein degradation via ubiquitin [10]. Deregulation of Jab1/CSN5 can lead to oncogenesis by inactivating numerous vital proteins, such as p27 [11]. Besides, increasing primary studies have indicated that Jab1/CSN5 is overexpressed in several human malignancies, such as breast cancer [12], lung carcinoma [13], and digestive system cancers [14, 15] and has a potential relationship with their prognosis. For example, high expression of CSN5 in colorectal cancer is associated with significantly shorter survival. It has also been reported that CSN5 regulates the deubiquitination and stability of PD-L1 [14]. Additionally, compound-15, an inhibitor of CSN5, can be used to destabilize PD-L1 to reduce tumor burden. Moreover, in gastric cancer specimens, increased levels of CSN5 protein have been related to lower overall survival (OS) [16]. Mechanistically, CSN5 is upstream of p14ARF and promotes the proliferation of gastric cancer cells. Besides, high expression of CSN5 is closely associated with TNM stage, tumor size and venous metastasis of hepatocellular carcinoma (HCC) patients [17]. The survival of HCC patients with low CSN5 expression is also significantly better compared to patients with the high expression. However, no reliable meta-analysis or systematic review determining the clinical significance of CSN5 in the assessment of digestive system cancers is currently available.

Therefore, in the present study, we conducted a meta-analysis to evaluate the potential significance of CSN5 as a novel marker for the prognosis of human digestive system malignancies. By comparing high and low expression levels of CSN5, an integrated meta-analysis was performed to search and analyze all published primary articles regarding the role of CSN5 in the prognosis prediction of digestive system malignancies.

## Methods

### Review protocol

The protocol for this review was defined in advance. The PRISMA checklist is provided in Table S1.

### Eligibility criteria

The following inclusion criteria were used to identify eligible studies: (1) human beings as study subjects and published articles with full-texts were prioritized; (2) patients diagnosed with digestive system cancer including colorectal cancer (CRC), gastric cancer (GC), hepatocellular carcinoma (HCC), pancreatic cancer (PC), gallbladder cancer (GBC) and esophageal squamous cell carcinoma (ESCC); (3) expression of CSN5 was measured in clinical samples of digestive system cancer; (4) the association between overall survival (OS) and recurrence-free survival (RFS) and CSN5 was evaluated; (5) at least two parameters were used to assess the correlation of CSN5 with clinicopathological characteristics; (6) Hazard Ratio (HR), Odds Ratio (OR) and their 95% confidence intervals (CIs) could be calculated with the data from the included articles. The most comprehensive or most recent data was analyzed for repetitive studies.

The study types included were: cross-sectional studies; prospective case-control studies; prospective cohort studies, retrospective cohort studies; clinical trials. Basic experimental studies with relevant data were also eligible for inclusion. No language, date, or publication status restrictions were applied.

The exclusion criteria included: (1) Letters to the editor, case reports, conference abstracts, meta-analyses, reviews, editorials and expert opinions. (2) Studies with overlapping data. (3) Studies with missing or insufficient data despite contacting original authors.

### Literature search strategy

The Web of Science, PubMed, EMBASE and CNKI databases were searched for related research published until April 11, 2021. The following keywords were used in the screen: “cancer or tumor or neoplasm or carcinoma” and “‘c-jun activation domain-binding protein 1’ or jab1 or ‘COP9 signalosome complex subunit 5 or Cop9 signalosome 5’ or CSN5 or COPS5”.

### Data extraction

All available data were independently extracted by two reviewers (Guo YH and Gao M). The evaluation of a third reviewer (Ma WJ) contributed to solving any disagreements over the information. For the survival analyses, the first author’s name, year of publication, country, methods used to detect CSN5, cut-off value, follow-up time, clinicopathological features and information utilized to calculated HRs and the 95% CIs were acquired. For each survival outcome, two methods were used to measure the HRs and the 95% CI: (1) directly available data of HRs and 95% CIs in the articles were retrived; (2) HRs and 95% CIs were indirectly obtained from the Kaplan-Meier survival curves via Engauge Digitizer version 4.1. This second method can lead to errors caused by variation. Additionally, we investigated the correlation between clinicopathologic parameters and CSN5 with ORs and 95% CIs.

### Quality assessment

Based on the Newcastle-Ottawa Scale (NOS), the quality of all eligible studies was independently investigated by two authors (Yao Y and Li JH). Scoring disagreements were settled through consensus. Every study was judged given three aspects: (I) group selection (four points, one score for each); (II) comparability (one point, up to two scores); and (III) assessment of either exposure or outcome (three points, one score for each). A high-quality study was determined by a score > 7.

### Statistical analyses

The inter-study heterogeneity was estimated by the χ^2^-based Cochrane Q-test. The definition of statistically significant heterogeneity was a χ^2^ *p* < 0.1 or an I^2^ index > 50%. If the inter-study heterogeneity was significant, a random-effect model was applied; otherwise, a fixed-effect model was performed. HRs and their 95% Cis were utilized to evaluate the significance of CSN5 in the survival of digestive system cancer patients. Lower CSN5 expression in patients with better survival was identified as HR > 1. In contrast, higher CSN5 expression in patients with a greater survival was recognized as HR < 1. Additionally, we evaluated the connection between CSN5 expression and clinicopathologic parameters using ORs and 95% CIs. Moreover, we performed a subgroup-analysis according to the tumor types when the data was available for analysis.

The Stata 11.0 Software (Stata Corporation, College Station) and the Revman 5.3 Software (Revman, the Cochrane Collaboration) were used for make all pooled analyses. To inspect the stability of our assessment, a sensitivity analysis was performed successively omitting one study at a time. The Begg’s funnel plot, as well as the Egger’s test, were used to evaluate potential publication bias, and a *p*-value < 0.05 was considered statistically significant.

## Results

### Search results

**Fig. 1:**
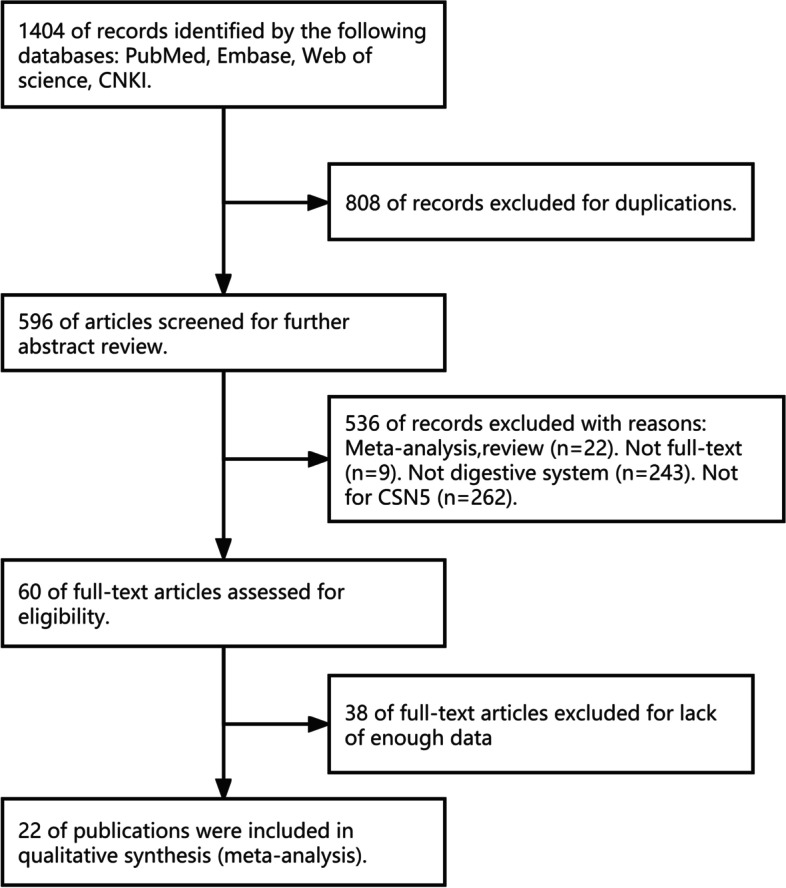
Flowchart of the study’s search and literature selection

After searching several international databases, 1404 articles were initially included. Then, 808 duplicates were excluded after analyzing the titles or abstracts. Next, 536 articles –reviews, studies that were not about CSN5, digestive system or full-texts–were excluded. Another 38 records were further excluded by screening the full texts, since they did not present sufficient data for analysis. Finally, the remaining 22 studies were used for analysis. The selection process is described in Fig. 1.

## Characteristics of the included studies

The principal features of the included studies are listed in Table 1. The studies analyzed were published between 2008 and 2020 the sum of patients reached 2193 (range: 40–286 for each study). All studies included were performed in Asian countries: 21 in China, and one in Japan. The Kaplan-Meier curves were used to indirectly calculate the HRs and 95% CIs, due to the absence of these parameters in some articles.

**Table 1:** Characteristics of studies included in the meta-analysis

First author, year	nation	Cancer type	Case number (High/Low)	Cut-off value	Detection method	Outcome	Follow-up time
Liu C,2020 [14]	China	CRC	189 (92/97)	Positive cells: +	IHC	OS	> 140 months
Wang L,2020 [16]	China	GC	90 (55/35)	Score = 8	IHC	OS	> 70 months
Zhou R,2018 [18]	China	CRC	116 (69/47)	Positive cells: +	IHC	OS	> 125 months
Shen Q,2020 [19]	China	ESCC	124 (65/59)	NA	IHC	OS	> 60 months
Pan YB,2017 [20]	China	CRC	286 (143/143)	NA	cDNA	RFS	192 months
Mao LX,2019 [21]	China	PC	106 (70/36)	NA	IHC	NA	NA
Kugimiya N,2017 [22]	Japan	CRC	50 (17/33)	ROC	RT-PCR	RFS	> 38 months
Liu HL,2018 [17]	China	HCC	102 (73/29)	NA	IHC	OS	> 80 months
Wang Y,2014 [23]	China	HCC	67 (41/26)	Score = 3	IHC	OS	60 months
Hsu MC,2008 [24]	China	HCC	99 (37/62)	Staining color: T = N	IHC	NA	NA
Chen L,2010 [15]	China	HCC	76 (43/33)	Positive cells = 69%	IHC	OS	60 months
Wang F,2009 [25]	China	ESCC	90 (75/15)	Positive cells = 10%	IHC	OS	60 months
Zheng L,2016 [26]	China	ESCC	187 (122/65)	Positive cells = 50%	IHC	OS	> 45 months
Guo ZQ,2014 [27]	China	CRC	80 (66/14)	Positive cells = 30%	IHC	NA	NA
Yang F,2013 [28]	China	GC	80 (57/23)	Score = 1	IHC	NA	NA
Zhang SW,2014 [29]	China	CRC	94 (81/13)	Score = 1	IHC	NA	NA
Cao Y,2013 [30]	China	HCC	40 (28/12)	Positive cells = 25%	IHC	NA	NA
Yang SH,2013 [31]	China	CRC	74 (60/74)	Score = 1	IHC	OS	60 months
Shi H,2010 [32]	China	ESCC	60 (47/13)	Positive cells = 25%	IHC	NA	NA
Gu GJ,2017 [33]	China	GBC	65 (39/26)	Score = 3	IHC	NA	NA
Zhang LY,2011 [34]	China	ESCC	58 (37/21)	Positive cells = 25%	IHC	NA	NA
Li S,2012 [35]	China	GC	60 (43/17)	Score = 1	IHC	OS	> 60 months

### Survival analysis

**Fig. 2:**
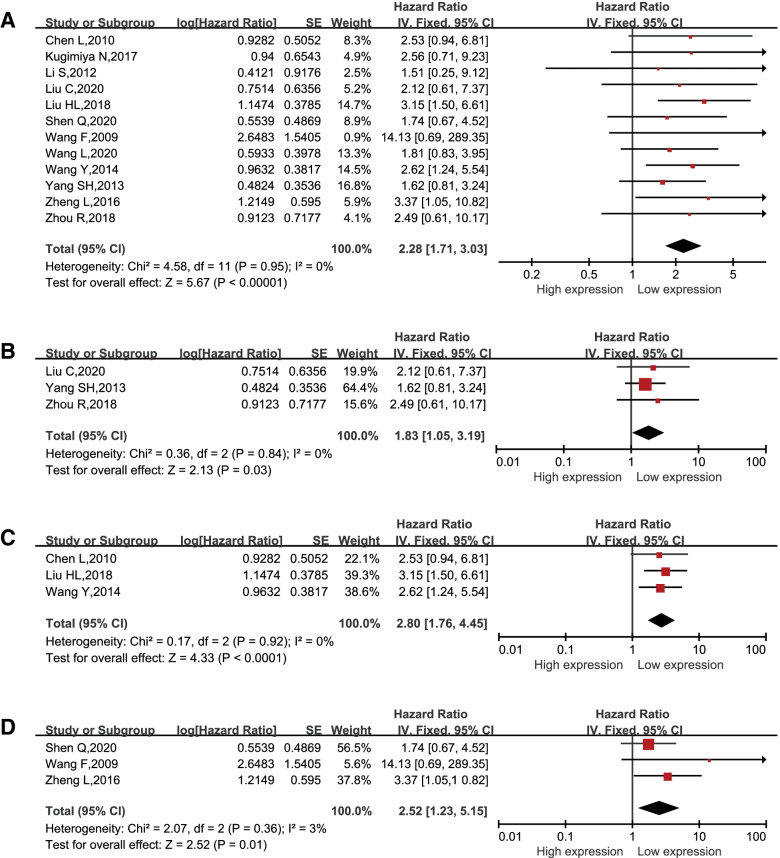
Forest plot of Hazard Ratios (HRs). Overall Survival (OS) for **A** all digestive system cancers; **B** CRC, **C** HCC, and **D** ESCC

After a pooled analysis of 22 studies comprehending 2193 patients, a combined HR of 2.28 (95% CI: 1.71–3.03; *p* < 0.00001; Fig. 2A) was determined to verify the significant association between poor OS of digestive system carcinomas and high expression of CSN5. Then, a fixed-effects model was applied since no significant heterogeneity was detected (χ^2^ = 0.36; freedom degrees = 11; *p* = 0.95; I^2^ = 0%). The subgroup analysis regarding the relation between CSN5 expression and OS in tumor types indicated that high expression of CSN5 was correlated with poor OS in CRC (HR = 1.83, 95% CI: 1.05–3.19; *p* = 0.03; Fig. 2B). Additionally, CSN5 overexpression was also clearly related to poor OS in HCC (HR = 2.80, 95% CI: 1.76–4.45; *p* < 0.00001; Fig. 2C). Moreover, a worse OS was detected for ESCC patients with high CSN5 expression (HR = 2.52, 95% CI: 1.23–5.15; *p* = 0.01; Fig. 2D). However, we did not detect a significant correlation between CSN5 expression and RFS (Figure S1).

### Association of CSN5 expression with clinical parameters

**Fig. 3:**
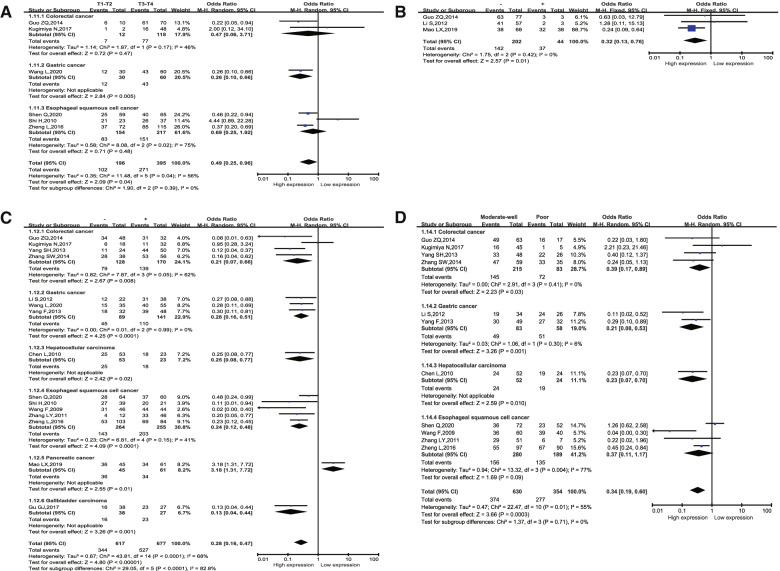
Forrest plot of OR. **A** invasion depth; **B** distant metastasis; **C** lymphatic metastasis; **D** differentiation degree

The correlation analysis for the clinicopathologic features and CSN5 level is presented in Table 2. High expression of CSN5 was significantly associated with poorer invasion depth (OR = 2.16, 95% CI: 1.49–3.13, *p* < 0.0001; Fig. 3A and Table S2), positive distant metastasis (OR = 3.37, 95% CI: 1.40–8.09, *p* = 0.007; Fig. 3B), positive lymphatic metastasis (OR = 3.14, 95% CI: 2.42–4.07, *p* < 0.00001; Fig. 3C and Table S3), and poorer differentiation degree (OR = 2.61, 95% CI: 1.90–3.60, *p* < 0.00001; Fig. 3D and Table S4). However, the levels of CSN5 were not significantly correlated with age, gender, tumor stage, tumor size or vascular invasion (Table 2, Figure S2 and Table S5). In the tumor-types subgroup analysis, patients with positive lymphatic metastasis in the CRC (OR = 4.53, 95% CI: 2.36–8.70, *p* = < 0.00001), GC (OR = 3.52, 95% CI: 1.97–6.30, *p* < 0.0001) and ESCC (OR = 4.13, 95% CI: 2.68–6.35, *p* < 0.00001) groups presented a correlation with high CSN5 expression. A correlation was also detected between CSN5 levelS of CSN5 and the differentiation degree in GC (OR = 4.96, 95% CI: 2.02–12.19, *p* = 0.0005), CRC (OR = 2.58, 95% CI: 1.21–5.51, *p* = 0.01) and ESCC (OR = 2.08, 95% CI: 1.37–3.15, *p* = 0.0005). However, the expression of CSN5 was not significantly correlated with invasion depth in CRC and ESCC. Finally, some tumor types were not available for analysis.

**Table 2:** Correlation of high CSN5 expression with clinicopathological parameters

Parameters	Studies	Case number	Pooled OR(95%CI)	P	Heterogeneity		Model	Publication bias
					I^2^	P		
Age	5	406	1.37 [0.89, 2.13]	0.16	0%	0.72	Fixed	0.462
Gender	17	1487	1.00 [0.78, 1.27]	0.97	34%	0.08	Fixed	0.343
TNM stage	9	721	0.81 [0.34, 1.91]	0.63	80%	< 0.00001	Random	0.536
Tumor size	8	749	0.83 [0.60, 1.16]	0.27	37%	0.14	Fixed	0.536
Invasion depth	6	591	0.49 [0.25, 0.96]	0.04	56%	0.04	Random	0.26
Lymphatic metastasis	15	1294	0.28 [0.16, 0.47]	< 0.00001	68%	< 0.00001	Random	0.701
Distant metastasis	3	246	0.32 [0.13, 0.76]	0.01	0%	0.42	Fixed	1
Differentiation degree	11	984	0.34 [0.19, 0.60]	0.0003	55%	0.01	Random	1
Venous invasion	4	322	1.11 [0.22, 5.53]	0.9	82%	0.0009	Random	1

### Publication Bias and sensitivity analysis

**Fig. 4:**
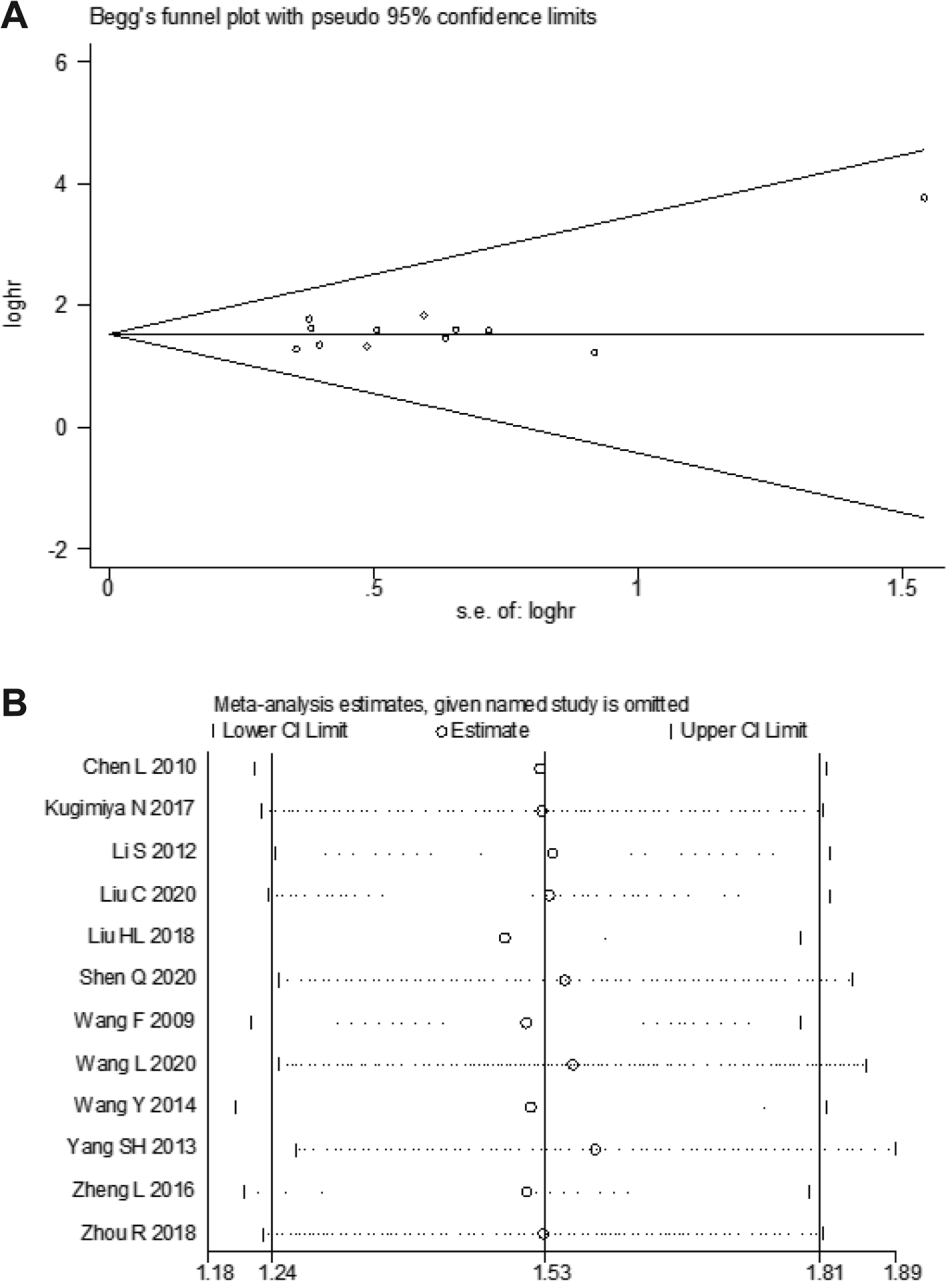
**A** Begg’s funnel plot for publication bias of OS; **B** Sensitivity analysis of OS

**Fig. 5:**
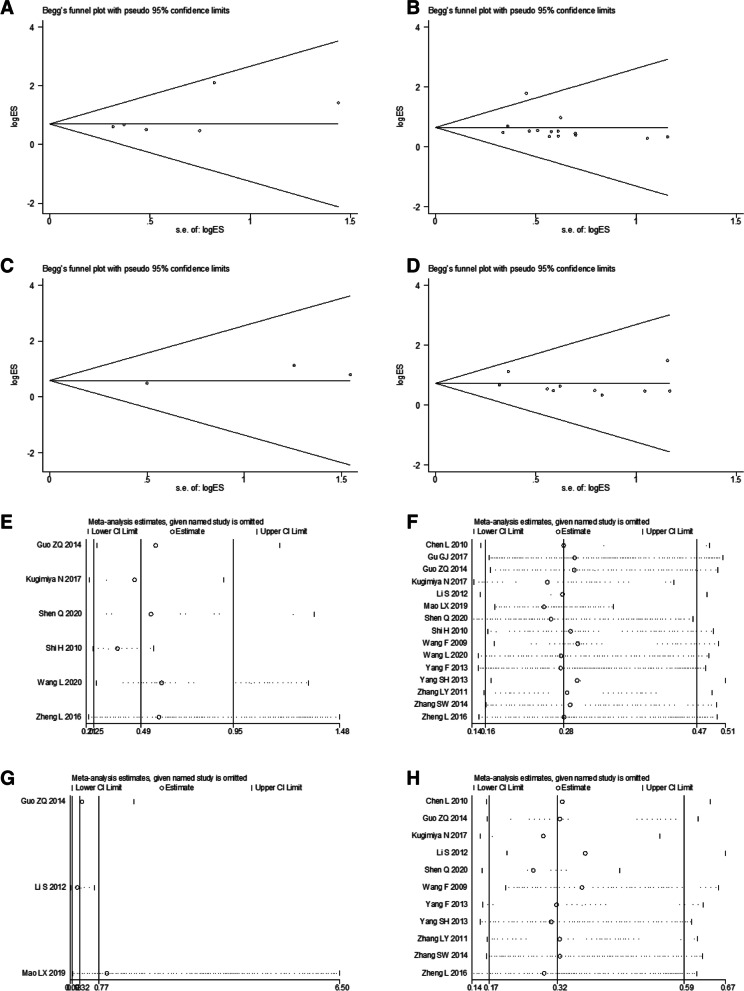
Begg’s funnel plot for publication bias of **A** invasion depth, **B** lymphatic metastasis, **C** distant metastasis and **D** differentiation degree. Sensitivity analysis of **E** invasion depth, **F** lymphatic metastasis, **G** distant metastasis, and **H** differentiation degree

To evaluate potential publication bias, Begg’s funnel plot and Egger’s test were conducted. We successively omitted one study at a time in the sensitivity analysis. The results of publication bias and sensitivity analysis within the included studies are presented in Figs. 4, S3, and S4. No significant publication bias was detected in the OS analysis (Egger’s test: *p* = 0.112). The sensitivity analysis results indicated the robustness and reliability of our estimates since the pooled results for OS were not be significantly altered by only one trial. The publication bias and sensitivity analysis results for RFS are shown in Figure S3. Moreover, the analysis for clinicopathological parameters demonstrated that no remarkable publication bias existed (Table 2). Regarding the sensitivity analysis, none of the pooled ORs for invasion depth, lymphatic metastasis, and differentiation degree were remarkably affected by eliminating any single study (Fig. 5). However, the sensitivity analysis of ORs for distant metastasis indicated a lack of stability. The publication bias and sensitivity analysis results of other clinicopathological characteristics are presented in Figure S4.

## Discussion

Several signal transduction pathways related to Jab1/CSN5 have been detected in oncological studies. Additionally, different tumoral features, such as cell cycle, cell apoptosis, and DNA damage repair have been found tobe correlated with Jab1/CSN5. Recently, various studies have been performed to investigate the diagnostic value of CSN5 to predict the survival of patients with digestive system cancers. However, the results remain inconclusive. To evaluate this question and evaluate the potential of CSN5 as a prognostic indicator, we implemented a meta-analysis of original articles to assess the value of CSN5 on the prognosis of digestive system cancer patients.

A total of 22 eligible articles were included, containing 2193 patients, to evaluate the correlation between CSN5 overexpression and survival outcomes as well as clinical parameters. We demonstrated the predictive role of high CSN5 levels for greater invasion depth, positive lymphatic metastasis, positive distant metastasis, poorer differentiation degree and poor survival. However, some results were not significant. First, in the subgroup survival analysis of gastric cancer, although it did not display a positive result, a positive tendency was detected. This might have occurred due to the inclusion of inadequate studies. Second, we did not detect a significant association between CSN5 expression and RFS in digestive system cancer patients. This result might be assigned to the deviation when the HRs were determined from the Kaplan–Meier curves in the included studies. Another cause might be the loss of sufficient research for pooled analysis since only 2 studies presented relevant information. Moreover, it is also possible that CSN5 did not influence the recurrence of digestive system cancers. Third, in the sensitivity analysis, the ORs of distant metastasis did not present sufficient robustness, which might be due to the different number of positive distant metastasis cases in all three studies. Fourth, certain clinicopathological characteristics did not show an obvious difference regarding the expression of CSN5, which might be related to the variation in cut-off values and the lack of studies with a large sample.

Furthermore, CSN5 can promote the transcription of MYC, an oncogenic transcription factor, target genes and induces its ubiquitination and turnover, thereby activating wound characteristics and inducing cancer cell proliferation and invasion [36], which might explain why CSN5 can affect tumor invasion depth. Moreover, Jab1/CSN5 can induce the degradation of two important downstream molecules, Smad4 and Smad7, and affect transforming growth factor-b (TGFb) signaling which is also influenced by various factors in the tumor microenvironment, and might contribute to lymphatic and distant metastasis [7, 37]. Additionally, Jab1/CSN5 has a direct interaction with p27, controlling cell proliferation through the inhibition of cyclin E-Cdk2, and mediates the shuttling of p27 between the nucleus and cytoplasm, inducing its degradation [11]. CSN5 can also promote cell proliferation and inhibit apoptosis by accelerating the nuclear export of p53 and inducing its degradation in a MDM2-mediated manner [38]. Overall, these findings might comprehend the underlying mechanisms of the influence of CSN5 on the poor OS of patients or the poor differentiation degree of tumors.

Previous studies have shown that CSN5 can be a novel potential therapeutic target against tumors. Recently, an inhibitor of Jab1/CSN5 (CSN5i-3) was used as a selective and orally available medicine, to inhibit the demethylating activity of CSN [39, 40]. The expression of CSN5 in tumor tissues is promising for future evaluations of CSN5 medications. Herein, we showed for the first time that high CSN5 expression in tumor tissues from digestive system cancer patients is related to poorer OS, demonstrating the clinical significance of CSN5 for the prediction of the survival of digestive system neoplasm patients.

However, our study also has some limitations. First, all included studies were published in Asia (21 in China and one in Japan). Therefore, our results might not apply to other ethnicities. Second, given that the HRs in all studies included could not be acquired, they were obtained from the Kaplan-Meier survival curves, which can lead to errors due to variation. Third, we detected some heterogeneity in the analysis of the clinicopathological features, which might be explained by the absence of standardization of quantification and cut-off thresholds. Moreover, the variation of detection method, with one study using clinical cDNA microarrays, another RT-PCR, and the remaining IHC, can result in errors, since the RNA levels of *CSN5* in tumor cells might differ from its protein level. However, even when we eliminated the two RNA studies (cDNA microarrays and RT-PCR), the results remained the same. Fourth, each of the included studies used different reference genes. Some studies used GAPDH and others beta-actin, which might also introduce errors. Moreover, GAPDH or β-actin have been considered constant, but, recently it has been shown that they can fluctuate under different tissue conditions [41]. Therefore, they might not be ideal for housekeeping genes. Fifth, the current study was not registered and might have a small bias. Nevertheless, we still strictly followed the steps of systematic evaluation. Finally, the conclusion drawn from our study need to be further confirmed by additional studies. Overall, these findings suggested that further detailed clinic studies with a uniform assessment assay should be performed to elucidate the prognostic value of CSN5 in digestive system neoplasm.

## Conclusion

The current meta-analysis demonstrated that high levels of CSN5 were correlated to poorer survival in digestive system neoplasm patients. The prognostic value of CSN5 can be further confirmed in other systemic tumors. Furthermore, the high expression of CSN5 was also related to some clinical features of malignancies. Finally, our results indicated that CSN5 had the potential to be an effective prognostic biomarker and a therapeutic target for digestive system tumors.

## Supplementary Information

**Additional file 1.**

**Additional file 2.**

**Additional file 3.**

**Additional file 4.**

**Additional file 5.**

**Additional file 6.**

**Additional file 7.**

**Additional file 8.**

**Additional file 9.**

## Data Availability

The datasets used and/or analyzed during the current study are available from the corresponding author on reasonable request.

## Abbreviations

CSN5: COP9 signalosome subunit 5
HR: Hazard Ratio
CI: Confidence interval
OS: Overall survival
RFS: Recurrence free survival
OR: Odds Ratio
CRC: Colorectal cancer
GC: Gastric cancer
HCC: Hepatocellular carcinoma
PC: Pancreatic cancer
GBC: Gallbladder cancer
ESCC: Esophageal squamous cell carcinoma
