# Supplementary figures and images for: Prognostic value of CSN5 in patients with digestive system cancers: a systematic review and meta-analysis
Source: BMC Cancer. 2022 Jul 23;22:812. doi: 10.1186/s12885-022-09867-9 (PMC9308938; doi:10.1186/s12885-022-09867-9)

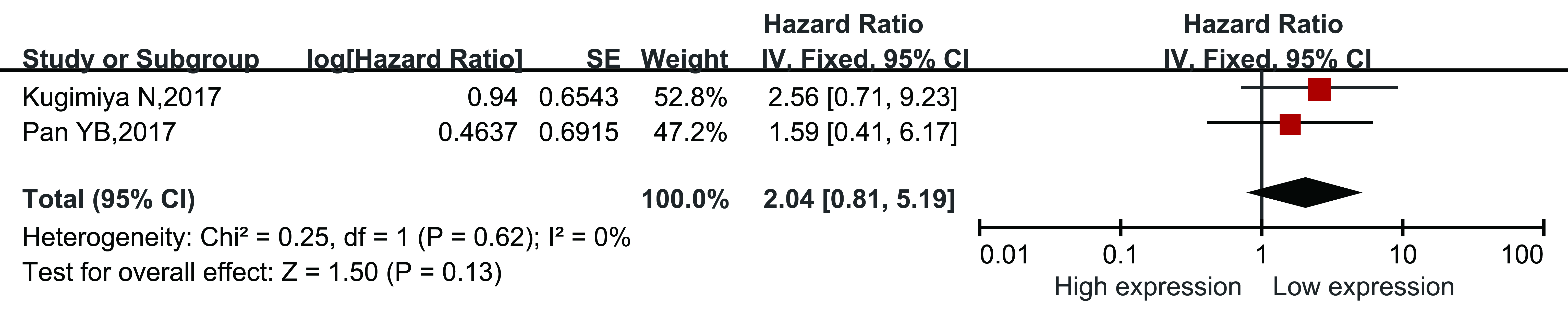

Supplement: Supplementary file 1 — Additional file 1. [file 12885_2022_9867_MOESM1_ESM.jpg]

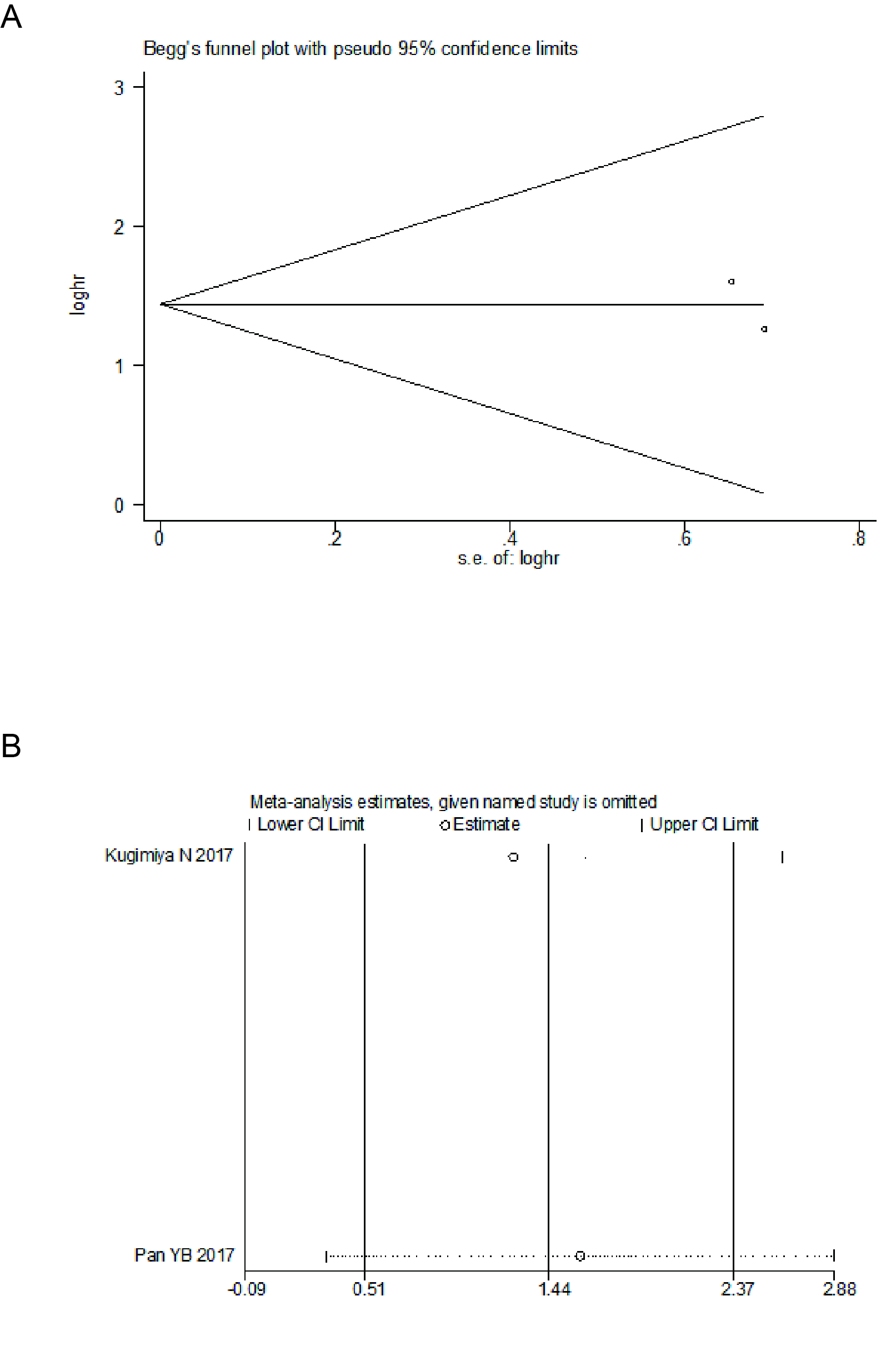

Supplement: Supplementary file 3 — Additional file 3. [file 12885_2022_9867_MOESM3_ESM.jpg]

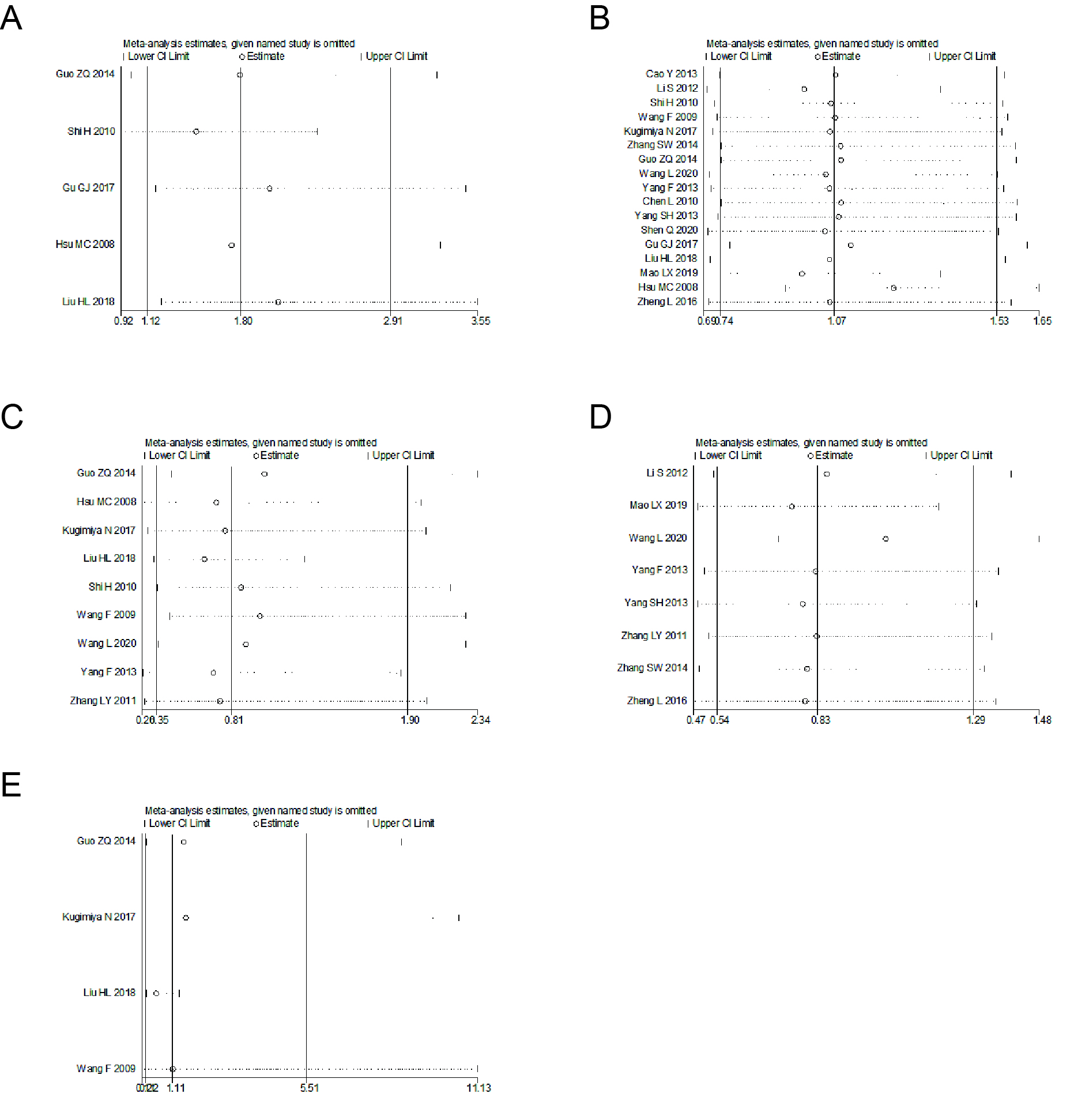

Supplement: Supplementary file 4 — Additional file 4. [file 12885_2022_9867_MOESM4_ESM.jpg]
