# Supplementary material for: Prognostic value of CSN5 in patients with digestive system cancers: a systematic review and meta-analysis
Source: BMC Cancer. 2022 Jul 23;22:812. doi: 10.1186/s12885-022-09867-9 (PMC9308938; doi:10.1186/s12885-022-09867-9)
Supplement: Supplementary file 6 — Additional file 6. [file 12885_2022_9867_MOESM6_ESM.docx]

|  | T3-T4 | | |  | T1-T2 | | | Weight | Pooled OR(95%CI) | P | Heterogeneity | |
| --- | --- | --- | --- | --- | --- | --- | --- | --- | --- | --- | --- | --- |
|  | High expression | Low expression | Total |  | High expression | Low expression | Total |  |  |  | I^2^ | P |
| Colorectal cancer | 77 | 41 | 118 |  | 6 | 4 | 10 | 7.1% | 2.56 [0.69, 9.51] | 0.16 | 46% | 0.17 |
| Gastric cancer | 43 | 17 | 60 |  | 12 | 18 | 30 | 12.3% | 3.79 [1.51, 9.53] | 0.005 | NA | NA |
| Esophageal squamous cell cancer | 151 | 66 | 217 |  | 83 | 71 | 154 | 80.5% | 1.87 [1.21, 2.88] | 0.004 | 75% | 0.02 |
|  |  |  |  |  |  |  |  |  |  |  |  |  |
| Total | 271 | 124 | 395 |  | 102 | 94 | 196 | 100% | 2.16 [1.49, 3.13] | <0.0001 | 56% | 0.04 |

**Table S2 – Subgroup analysis of invasion depth**
