# Supplementary material for: Prognostic value of CSN5 in patients with digestive system cancers: a systematic review and meta-analysis
Source: BMC Cancer. 2022 Jul 23;22:812. doi: 10.1186/s12885-022-09867-9 (PMC9308938; doi:10.1186/s12885-022-09867-9)
Supplement: Supplementary file 7 — Additional file 7. [file 12885_2022_9867_MOESM7_ESM.docx]

|  | Lymphatic metastasis | | | | No lymphatic metastasis | | | | | | Weight | | Pooled OR(95%CI) | P | | Heterogeneity | | | |  |
| --- | --- | --- | --- | --- | --- | --- | --- | --- | --- | --- | --- | --- | --- | --- | --- | --- | --- | --- | --- | --- |
|  | High expression | Low expression | Total | | High expression | Low expression | | Total | |  | |  | | |  | | I^2^ | | P | |
| Colorectal cancer | 139 | 31 | | 170 | 79 | | 49 | | 128 | | 14.2% | | 4.53 [2.36, 8.70] | <0.00001 | | 62% | | 0.05 | |  |
| Gastric cancer | 110 | 31 | | 141 | 45 | | 44 | | 89 | | 17.7% | | 3.52 [1.97, 6.30] | <0.0001 | | 0% | | 0.99 | |  |
| Hepatocellular carcinoma | 18 | 5 | | 23 | 25 | | 28 | | 53 | | 4.9% | | 4.03 [1.30, 12.46] | 0.02 | | NA | | NA | |  |
| Esophageal squamous cell cancer | 203 | 52 | | 255 | 143 | |  | | 264 | | 32.8% | | 4.13 [2.68, 6.35] | <0.00001 | | 41% | | 0.15 | |  |
| Pancreatic cancer | 34 | 27 | | 61 | 36 | | 9 | | 45 | | 27.4% | | 0.31 [0.13, 0.77] | 0.01 | | NA | | NA | |  |
| Gallbladder carcinoma | 23 | 4 | | 27 | 16 | | 22 | | 38 | | 2.9% | | 7.91 [2.28, 27.37] | 0.001 | | NA | | NA | |  |
|  |  |  | |  |  | |  | |  | |  | |  |  | |  | |  | |  |
| Total | 271 | 124 | | 395 | 344 | | 273 | | 617 | | 100% | | 0.49 [0.25, 1.96] | <0.00001 | | 68% | | <0.001 | |  |

**Table S3 – Subgroup analysis of lymphatic metastasis**
