# Supplementary material for: Prognostic value of CSN5 in patients with digestive system cancers: a systematic review and meta-analysis
Source: BMC Cancer. 2022 Jul 23;22:812. doi: 10.1186/s12885-022-09867-9 (PMC9308938; doi:10.1186/s12885-022-09867-9)
Supplement: Supplementary file 8 — Additional file 8. [file 12885_2022_9867_MOESM8_ESM.docx]

|  | Poor | | |  | Moderate-well | | | Weight | Pooled OR(95%CI) | P | Heterogeneity | |
| --- | --- | --- | --- | --- | --- | --- | --- | --- | --- | --- | --- | --- |
|  | High expression | Low expression | Total |  | High expression | Low expression | Total |  |  |  | I^2^ | P |
| Colorectal cancer | 72 | 11 | 83 |  | 145 | 70 | 215 | 18.9% | 2.58 [1.21, 5.51] | 0.01 | 0% | 0.41 |
| Gastric cancer | 51 | 7 | 58 |  | 49 | 34 | 83 | 10.0% | 4.96 [2.02, 12.19] | 0.0005 | 6% | 0.30 |
| Hepatocellular carcinoma | 19 | 5 | 24 |  | 24 | 28 | 52 | 6.4% | 4.43 [1.44, 13.67] | 0.01 | NA | NA |
| Esophageal squamous cell cancer | 135 | 54 | 189 |  | 156 | 124 | 280 | 64.7% | 2.08 [1.37, 3.15] | 0.0005 | 77% | 0.004 |
|  |  |  |  |  |  |  |  |  |  |  |  |  |
| Total | 271 | 124 | 395 |  | 102 | 94 | 196 | 100% | 2.61 [1.90, 3.60] | 0.04 | 55% | 0.01 |

**Table S4 – Subgroup analysis of differentiation degree**
