# Supplementary material for: Prognostic value of CSN5 in patients with digestive system cancers: a systematic review and meta-analysis
Source: BMC Cancer. 2022 Jul 23;22:812. doi: 10.1186/s12885-022-09867-9 (PMC9308938; doi:10.1186/s12885-022-09867-9)
Supplement: Supplementary file 9 — Additional file 9. [file 12885_2022_9867_MOESM9_ESM.docx]

|  | Male | | |  | Female | | | Weight | Pooled OR(95%CI) | P | Heterogeneity | |
| --- | --- | --- | --- | --- | --- | --- | --- | --- | --- | --- | --- | --- |
|  | High expression | Low expression | Total |  | High expression | Low expression | Total |  |  |  | I^2^ | P |
| Colorectal cancer | 129 | 59 | 188 |  | 78 | 32 | 110 | 19.7% | 0.84 [0.48, 1.49] | 0.56 | 0% | 0.93 |
| Gastric cancer | 113 | 50 | 163 |  | 42 | 35 | 77 | 12.6% | 1.98 [1.12, 3.49] | 0.02 | 55% | 0.11 |
| Hepatocellular carcinoma | 119 | 28 | 228 |  | 62 | 27 | 89 | 26.3% | 0.58 [0.34, 1.00] | 0.05 | 65% | 0.04 |
| Esophageal squamous cell cancer | 234 | 111 | 345 |  | 75 | 41 | 116 | 25.6% | 1.16[0.74, 1.84] | 0.52 | 0% | 0.97 |
| Pancreatic cancer | 40 | 20 | 60 |  | 30 | 16 | 46 | 8.6% | 1.07 [0.47, 2.40] | 0.88 | NA | NA |
| Gallbladder carcinoma | 11 | 11 | 22 |  | 28 | 15 | 43 | 7.2% | 0.54 [0.19, 1.52] | 0.24 | NA | NA |
|  |  |  |  |  |  |  |  |  |  |  |  |  |
| Total | 646 | 360 | 1006 |  | 315 | 166 | 481 | 100% | 1.00 [0.78, 1.27] | 0.97 | 34% | 0.08 |

**Table S5 – Subgroup analysis of gender**
